# Orexin, Sleep, and Cognition in Alzheimer Disease: Non-REM Oscillatory Activity and Neural Resilience

**DOI:** 10.1212/WNL.0000000000218307

**Published:** 2026-07-14

**Authors:** Arsenio Paez, Gerard Piñol-Ripoll, Anna Carnes-Vendrell, Farida Dakterzada, Ferran Barbé, Henrik Zetterberg, Thien Thanh Dang-Vu

**Affiliations:** 1 Nuffield Department for Surgical Sciences, University of Oxford, United Kindom;; 2 Centre de Recherche de l'Institut Universitaire de Gériatrie de Montréal (CRIUGM), Centre Intégré Universitaire de Santé et Services Sociaux du Centre-Sud-de-l'île-de-Montréal, Canada;; 3 Sleep, Cognition and Neuroimaging Laboratory, Concordia University, Montreal, Canada;; 4 Bouvé College of Health Sciences, Northeastern University, Boston, MA;; 5 Unitat de Trastorns Cognitius, Cognition and Behavior Study Group, Hospital Universitari Santa Maria, Universitat de Lleida, IRBLleida, Spain;; 6 Alzheimer's Disease and Other Cognitive Disorders Unit, Neurology Service, Hospital Clínic de Barcelona, Fundació de Recerca Clínic–Institut d'Investigacions Biomèdiques August Pi i Sunyer (IDIBAPS), Spain;; 7 Universitat de Lleida, Spain;; 8 Institut de Recerca Biomèdica de Lleida–Fundació Dr. Pifarré (IRBLleida), Grup d'Estudi de la Cognició i la Conducta, Spain;; 9 Translational Research in Respiratory Medicine (TRRM), Hospital Universitari Arnau de Vilanova-Santa Maria, Biomedical Research Institute of Lleida (IRBLleida), Spain;; 10 Department of Psychiatry and Neurochemistry, Institute of Neuroscience and Physiology, Sahlgrenska Academy, University of Gothenburg, Mölndal, Sweden;; 11 Clinical Neurochemistry Laboratory, Sahlgrenska University Hospital, Mölndal, Sweden;; 12 Department of Neurodegenerative Disease, UCL Institute of Neurology, London, United Kingdom;; 13 UK Dementia Research Institute at UCL, London, United Kingdom;; 14 Department of Pathology and Laboratory Medicine, University of Wisconsin School of Medicine and Public Health, Madison;; 15 Wisconsin Alzheimer's Disease Research Center, University of Wisconsin School of Medicine and Public Health, University of Wisconsin-Madison;; 16 Hong Kong Center for Neurodegenerative Diseases, InnoHK, China; and; 17 Centre for Brain Research, Indian Institute of Science, Bangalore, India.

## Abstract

**Background and Objectives:**

Sleep-wake dysregulation and elevated CSF orexin have been implicated in Alzheimer disease (AD). Sleep spindles (SPs) and slow oscillations (SOs) are linked to cognition and neurodegeneration; however, their relationship with CSF orexin concentrations in symptomatic AD has not been characterized. We investigated whether nonrapid eye movement (NREM) SP-SO activity is associated with CSF orexin and whether these oscillatory features moderate associations between orexin, cognition, neuropsychiatric symptom severity, and AD biomarkers.

**Methods:**

This prospective observational cohort study was conducted at a tertiary memory clinic in Lleida, Spain. Individuals aged ≥60 years with biomarker-confirmed mild-to-moderate AD (National Institute on Aging-Alzheimer's Association criteria) underwent overnight polysomnography and morning CSF sampling. SP and SO were detected using validated automated algorithms with independent verification and visual quality control. CSF was assayed for orexin-A, amyloid-β42 (Aβ42), phosphorylated tau181 (pTau181), total tau, and YKL-40. Cognitive performance Alzheimer’s Disease Assessment Scale–Cognitive Subscale ([ADAS-Cog], Mini-Mental State Examination (MMSE), California verbal learning test, ROCF) and neuropsychiatric symptoms (NPI) were assessed longitudinally over 36 months. Associations were examined using generalized linear models with robust estimators adjusted for age, sex, Aβ42, and apnea-hypopnea index. Multiple comparisons were controlled using false discovery rate correction. Interaction terms assessed moderation effects.

**Results:**

Sixty participants (30 women; mean age 74.7 years) were included. Longer SO duration and higher SP density and power were associated with lower CSF orexin concentrations (SP density: β = −187.37 pg/mL, 95% CI −344.93 to −29.80). Orexin was not associated with global sleep continuity metrics. Higher CSF orexin concentrations were associated with worse global cognition (ADAS-Cog: β = 0.014, 95% CI 0.003–0.024; MMSE: β = −0.01, 95% CI −0.011 to −0.004) and greater neuropsychiatric symptom severity (NPI at: β = 0.03, 95% CI 0.011–0.041). Higher orexin was also associated with higher pTau181 (β = 0.11, 95% CI 0.04–0.19), total tau, and YKL-40 (β = 0.37, 95% CI 0.17–0.57). Significant orexin × SP-SO interactions were observed, such that greater oscillatory activity attenuated the adverse associations between orexin and cognitive outcomes, independent of Aβ42 and tau.

**Discussion:**

In biomarker-confirmed AD, NREM SP and SO activity are associated with CSF orexin concentrations and moderate associations between orexin and longitudinal cognitive and neuropsychiatric outcomes. Limitations include the observational design and absence of a comparator group, precluding causal inference and limiting contextualization relative to normal aging. NREM oscillatory metrics and orexin concentrations may represent complementary physiologic markers for disease monitoring and therapeutic targeting.

**Trial Registration Information:**

Role of Hypoxia and Sleep Fragmentation in AD; ClinicalTrials.gov Identifier: NCT02814045.

## Introduction

Sleep disturbances affect up to two-thirds of persons with Alzheimer disease (AD) and are increasingly recognized as both consequences and contributors to AD progression.^1-3^ Experimental and human evidence links insufficient or fragmented sleep to impaired glymphatic clearance of β-amyloid (Aβ) and tau, proteins central to AD pathogenesis, and to altered synaptic homeostasis, and degradation of nonrapid eye movement (NREM) oscillatory features. These features, specifically sleep spindles (SP) and slow oscillations (SO), support memory consolidation and cortical network stability, and their disruption in people with AD is increasingly recognized as a physiologic marker of cognitive decline and a potential therapeutic target.^1,3-5^

Orexin (hypocretin) is a neuropeptide that may also influence AD pathophysiology.^6,7^ Produced in the lateral hypothalamus, orexin-A and B promote arousal, stabilize sleep-wake transitions, and regulate rapid eye movement (REM) sleep.^8,9^ Orexinergic projections extend widely across the brain, including regions critical for cognition such as the hippocampus and prefrontal cortex.^10^ Dysregulation of the orexin system contributes to sleep-wake disorders such as narcolepsy and insomnia.^11^ Beyond sleep-wake regulation, orexin influences cognition and emotion through modulation of cortical, limbic, and monoaminergic circuits that underlie attention, learning, memory, mood, and stress regulation.^12^ Dysregulated orexin signaling is associated with apathy, depression, and anxiety and may contribute to neuropsychiatric symptoms in dementia.^13^ However, few studies have examined whether orexinergic activity relates to cognitive decline or neuropsychiatric symptom severity in people with AD.

Orexin is also emerging as an important treatment target in sleep disorders. Orexin receptor antagonists are now approved for the treatment of insomnia disorder.^11^ It is important that dual orexin receptor antagonists (DORAs) have also shown cognitive and behavioral benefits in AD models, underscoring the therapeutic relevance of orexin modulation in neurodegeneration.^14^ Given the availability of orexin-targeting therapies and the high prevalence of sleep disruption in AD, understanding how orexinergic activity interacts with sleep neurophysiology has direct clinical relevance for prognostic monitoring and treatment development. Both orexin and NREM sleep microarchitecture can be objectively quantified using established clinical and research tools, facilitating translation of these physiologic findings into prognostic and interventional frameworks.

Important evidence gaps remain regarding the interplay between orexin and AD.^15^ Evidence on CSF orexin concentrations in people with AD remains inconsistent. Some studies report higher CSF orexin concentrations in people with mild cognitive impairment and AD,^9,10,16,17^ often correlating with Aβ and phosphorylated tau.^7,18^ Others, including meta-analyses, find no group differences or biomarker associations relative to cognitively healthy older adults.^15,18^ Findings on sex-based differences are similarly inconsistent.^9,19^ These discrepancies likely reflect methodological and clinical heterogeneity.^15^

Given orexin's central role in arousal, sleep likely represents a critical intermediary linking orexin, cognition, and neurodegeneration. Omitting sleep from analyses may partly explain prior inconsistencies. Elevated CSF orexin has been associated with sleep and circadian rhythm disruption in older adults and people with AD,^9^ but most studies have relied on subjective sleep reports or broad sleep measures such as total sleep time (TST) or sleep efficiency (SE). Prior studies have examined CSF orexin in relation to sleep macroarchitecture and clinical features in AD,^16^ and emerging work has linked NREM oscillatory activity to AD biomarkers and cognition.^20,21^ In addition, prior analyses from this cohort have characterized associations between sleep microarchitecture and neurodegenerative biomarkers.^20^ However, no studies to our knowledge have examined the relationship between CSF orexin and NREM sleep microarchitecture, or their combined effects on longitudinal clinical outcomes, in biomarker-confirmed symptomatic AD.

Whether orexin interacts with these NREM features, and how these interactions affect cognition and behavior in people with AD, remains unknown. This is an important gap, given established associations between SP and SO with sleep continuity,^22^ cognition,^20^ and neurodegeneration.^20,21^ Clarifying these interactions may identify physiologic markers and new therapeutic targets for sleep-based or orexin-modulating interventions in people with AD.

To address these gaps, we investigated relationships among CSF orexin concentrations, objectively measured NREM sleep microarchitecture, cognition, neuropsychiatric symptom severity, and CSF biomarkers of neurodegeneration in a prospective cohort of individuals with mild-to-moderate AD. We hypothesized that

1. Greater SP and SO activity are associated with lower CSF orexin concentrations.
2. Higher CSF orexin concentrations would be associated with poorer cognitive performance.
3. Higher CSF orexin concentration would be associated with greater neurodegeneration and neuroinflammation (AD biomarkers).
4. SP and SO activity would attenuate the adverse effects of orexin on cognition.

In secondary analyses, we examined sex-based differences in orexin, given uncertainty surrounding sex effects in people with AD and known sex-based influences on NREM oscillations, and explored associations among orexin, SP-SO activity, and neuropsychiatric symptom severity.

## Methods

### Study Design and Participants

We collected data between November 2014 and November 2017 in a prospective cohort study investigating sleep and cognitive decline in people with AD.^3^ Detailed participant eligibility, recruitment, and data collection have been published previously.^3^ Participants were recruited consecutively and prospectively from the Unitat de Trastorns Cognitius (Cognitive Disorders Unit) of Hospital Universitari Santa Maria (Lleida, Spain), a tertiary referral memory clinic specializing in the evaluation and management of cognitive impairment and neurodegenerative disorders. Referrals are typically made by neurologists, geriatricians, and primary care physicians.

Eligible individuals were aged ≥60 years and had mild-to-moderate AD diagnosed following National Institute on Aging-Alzheimer's Association criteria.^23^ Exclusion criteria included the use of investigational drugs, beta-blockers, antidepressants, neuroleptics, or hypnotics within 15 days before overnight polysomnography (PSG).^3^ No participants were taking anticonvulsants, antipsychotics, anxiolytics, hypnotics, orexin antagonists, or benzodiazepine receptor agonists, which could alter sleep microarchitecture.

### Data Collection

#### Sleep and Biomarkers

**Figure 1:**
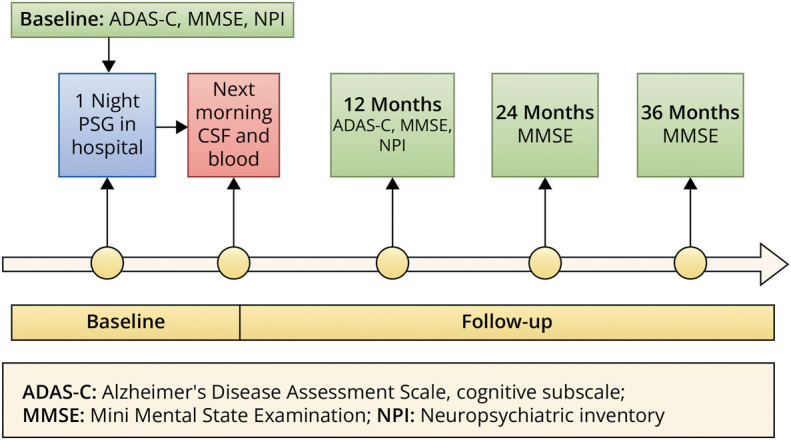
Study Methods: “Role of Hypoxia and Sleep Fragmentation in Alzheimer Disease” PSG = polysomnography.

At baseline, participants underwent a single overnight PSG, using a Philips Respironics Alice 6 LDx system with 34 EEG channels referenced to the mastoids and sampled at 512 Hz. EEG signals were visually scored by experienced sleep technicians (bandpass filter: 0.3–93.6 Hz) in 30-second epochs according to American Academy of Sleep Medicine criteria^24^ (Figure 1). All recordings underwent standard quality control procedures, and no PSG recordings were excluded because of technical failure.

The following morning, CSF was collected and analyzed for markers of neuroinflammation and neurodegeneration, including amyloid-β42 (Aβ42), phosphorylated tau181 (pTau181), total tau, and chitinase-3-like protein-1 (YKL-40), a marker of AD neuroinflammation, and neurofilament-light chain using commercially available ELISA kits, as previously described.^25^ CSF orexin concentrations were measured using radioimmunoassay using established protocols.^26^

#### Cognition and Neuropsychiatric Symptoms

Participants completed functional and neuropsychological assessments at baseline and 12-month follow-up visits at IRBLleida, including the Alzheimer’s Disease Assessment Scale–Cognitive Subscale (ADAS-Cog),^27^ California verbal learning test (CVLT), Rey-Osterrieth Complex Figure Test (ROCF), Cornell Scale for Depression in Dementia, and Neuropsychiatric Inventory (NPI).^28^ The Mini-Mental State Examination (MMSE)^29^ was administered at baseline, 12-, 24-, and 36-month follow-up visits.

#### Sleep EEG and Event Detection

Spindles and SO were detected on artifact (e.g., movement, flatlining, signal pops) free epochs during NREM2 and NREM3 using in-house software^30^ incorporating validated algorithms (spindles: Mölle et al., 2011^31^; SO: Staresina et al., 2015^32^).

Spindles were detected over central derivations (C3-A2, C4-A1) within a 9- to 16-Hz bandwidth^33^ and 0.5- to 3-second duration. This frequency range captured both slow (9–12 Hz) and fast (13–16 Hz) spindles, which decline with age and have been linked to cognitive impairment. Root mean square (RMS) amplitude was computed over a 0.2-second sliding window and smoothed with a 0.2-second moving average. Spindles were defined as events where RMS exceeded 1.5 standard deviations of the mean for 0.5–3.0 seconds.^31^

Slow oscillations were detected over frontal derivations (F3-A2, F4-A1) between 0.16 and 1.25 Hz with a zero-phase infinite impulse response bandpass filter, durations of 0.8–2.0 seconds. Following Staresina's criteria, the event amplitude threshold for SOs meeting duration criteria was amplitudes exceeding the 75th percentile of trough-to-peak amplitude between 2 positive-to-negative zero crossings.^32^

To ensure reliability, automated detections were independently verified by 2 trained investigators using identical detection parameters. All detected events were subsequently visually reviewed by 2 investigators to confirm accuracy and exclude artifacts or false positives. Extracted metrics included SP and SO count, density (per 30 seconds), duration, power (μV^2^), and SO peak-to-peak amplitude (μV), each previously linked to cognition^34^ and neurodegeneration in older adults.^21,35^

### Statistical Analyses

All statistical analyses were undertaken with the Stata 18.5.^36^ Descriptive statistics summarized demographics, sleep measures (continuous), biomarkers (continuous), and cognitive and neuropsychiatric outcomes (continuous). Distributional normality was examined with Shapiro-Wilk tests and histogram/kernel density plots. We calculated pTau181/Aβ42 and total tau/Aβ42 ratios, as they outperform individual biomarkers in predicting clinical and cognitive decline.^37^

Associations among SP and SO metrics, orexin, AD biomarkers (Aβ42, total tau, pTau181, pTau181/Aβ42, total tau/Aβ42), cognition (ADAS-Cog, MMSE, CVLT, ROCF), and neuropsychiatric symptoms (NPI) were examined using generalized linear models with Huber-White sandwich estimators to account for non-normal errors and heteroskedasticity in residual distributions. Longitudinal clinical outcomes (e.g., 12-, 24-, and 36-month measures) were analyzed in separate models at each time point, such that each model included 1 observation per participant and did not require a mixed-effects or repeated-measures framework. Models adjusted for age, sex, baseline Aβ42, and apnea-hypopnea index-AHI (continuous). Analyses of orexin with cognition or NPI in addition controlled for TST, given evidence for the association between sleep duration and both orexin and cognition.^8,11,38^ Additional sensitivity analyses were conducted adjusting for obstructive sleep apnea (OSA) severity (severe vs nonsevere; AHI ≥30 events/hour), modeled as a binary variable to preserve statistical power given the sample size.

Multiple comparisons were corrected using the Benjamini-Hochberg false discovery rate (FDR) with a prespecified threshold of q < 0.05. Multicollinearity among SP-SO features was checked with variance inflation factors (VIFs), all <2 (no to low correlation).^39^ There was no missing data.

Moderation effects were assessed using generalized linear models including interaction terms. Mediation effects were evaluated separately using Sobel-Goodman tests of indirect effects following the framework described by Preacher and Hayes.^40^ These analyses evaluated whether SP, SO, or orexin mediated or moderated associations with cognition or neuropsychiatric symptoms, adjusting for age, sex, AHI, and Aβ42 and pTau181, given their established associations with cognition in persons with AD.

### Standard Protocol Approvals, Registrations, and Participant Consents

The study was approved by the Institutional Review Board of Hospital Arnau de Vilanova de Lleida (approval number CE-1218) and conducted in accordance with the Declaration of Helsinki and its subsequent amendments. All participants provided written informed consent before participation. The study was registered at ClinicalTrials.gov (Identifier: NCT02814045).

### Data Availability

Anonymized data analyzed in this study will be made available to qualified researchers upon reasonable request, subject to ethical or legal considerations regulating participant data access.

## Results

Sixty participants (30 women) with a mean age of 74.7 (SD 5.0) years (Table 1) were eligible and completed the study. Participants had a median CSF Aβ42 of 516 pg/mL at baseline. No sex differences were observed in CSF Aβ42, tau, or tau/Aβ42 ratios; however, CSF orexin concentrations were significantly higher in women than in men (837 [41] pg/mL vs 673 [35] pg/mL; *p* = 0.004; overall mean = 757.3 [29] pg/mL). Mean TST was 260 minutes (SD 78.5; interquartile range 211.5–328.0), and median sleep efficiency (SE) was 67% (interquartile range, 48–80) (Table 1). TST was not significantly associated with CSF orexin concentrations or cognitive outcomes (all *p* > 0.20). No significant sex differences were found in TST, SE, or most NREM microarchitecture features, although women showed higher spindle density (per 30-second epoch) and power than men (eTable 1). Neuropsychiatric symptom severity and most cognitive measures did not differ by sex, except for higher ROCF long-term visual memory scores in men.

**Table 1:** Participants' Characteristics at Baseline

Sample	Male (n = 30)	Female (n = 30)	Total (n = 60)	*p* Value
Age	75.5 ± 5.0	74.0 ± 5.0	74.7 ± 5.0	0.26
Body mass index (BMI)	27 (24–29)	28 (24–32)	27 (24–32)	0.71
Depression, (%)	6 (20)	12 (40)	18 (30)	0.09
Diabetes, (%)	7 (23)	3 (10)	10 (17)	0.17
Education (≥ high school), (%)	5(17)	5(17)	10 (17)	1.00
0: No formal education	2 (7)	2 (7)	4(7)	
1. Primary school	23 (77)	23 (77)	46(77)	
2. High school	4 (13)	4 (13)	8 (13)	
3. University	1 (3)	1 (3)	2 (3)	
Smoking history, (%)	11(37)	2 (7)	41(68)	0.005^a^
0. Never	19 (63)	28 (93)	47 (78)	
1. Current	2 (7)	0 (0)	2 (3)	
2. Former (>6 mo ago)	9 (30)	2 (7)	11(18)	
Obstructive sleep apnea (OSA), (%)	24 (96)	23 (89)	47(78)	0.25
Apnea hypoxia index (n/hrTST), (%)	38.14 ± 23	29.24 ± 22.8	33.7 ± 23.14	0.14
0–4.9	1 (3)	2 (7)	3 (5)	
5–14.9	4 (13)	7 (23)	11 (18)	
15–30	9 (30)	10 (33)	19 (32)	
≥30	16 (53)	11 (37)	27 (45)	
AD drugs	26 (87)	27(90)	53 (88)	0.69
None	4 (13)	3 (10)	7 (12)	
Rivastigmine	9 (30)	9 (30)	18 (30)	
Donepezil	17 (57)	15 (50)	32 (53)	
Memantine	0	3 (10)	3 (6)	
CSF values-biomarkers (pg/mL)				
Beta-amyloid (Aβ42)	506 (417–609)	532 (398–627)	516 (411–618)	0.62
Phosphorylated tau (pTau181)	77 ± 4.4	84.07 ± 5.94	70.44 ± 6.32	0.12
Total tau	487 ± 288	599 ± 277	543 ± 285	0.16
PTau181/Aβ42 ratio	0.14 ± 0.07	0.17 ± 0.08	0.16 ± 0.74	0.25
Total-tau/Aβ42 ratio	0.87 (0.52–1.34)	1.10 (0.70–1.63)	0.96 (0.55–1.48)	0.27
Chitinase-3-like protein (YKL-40) (ng/mL)	289 (226.-413)	289 (195–379)	283 (225–389)	0.65
Orexin (pg/mL)	673 ± 35	838 ± 41	757 ± 29	0.00^a^
Cognition				
ADAS-cog total score	28 (26–32)	29 (25–31)	29 (25–31)	0.89
MMSE	23.4 ± 2.3	23.0 ± 2.4	23.2 ± 2.4	0.55
Neuropsychiatric				
Cornell scale (CSDD)	7 (2–11)	6 (3–11)	7 (3–11)	0.93
Neuropsychiatric index (NPI)	4 (0–11)	8 (3–13)	6 (2–12)	0.51

Abbreviations: AD = Alzheimer disease; ADAS-Cog = Alzheimer’s Disease Assessment Scale–Cognitive Subscale; MMSE = Mini-Mental State Examination.

SI conversion factors: To convert Aβ42, pTau181, total tau, orexin to mmol/L, multiply values by 0.022. To convert YKL-40 to mmol/L, multiply by 27.74.

a Statistically significant difference.

### Associations Between Sleep Oscillations and CSF Orexin

Spindle and SO features were associated with CSF orexin levels (Table 2). Longer SO duration and higher SP density, duration, and power were associated with lower orexin concentrations, although not all associations remained significant after FDR correction. By contrast, orexin was not significantly related to global sleep continuity metrics including TST, SE, wake after sleep onset, or sleep-onset latency.

**Table 2:** GLM Regression Results: CSF Orexin and AD Biomarkers, Cognition, SP, SO Activity

Orexin associations with AD biomarkers (pg/mL)	β coeff	SE	*p* Value	95% CI lower	Upper
Amyloid-beta 42 (Aβ42)	0.03	0.11	0.02^a^	0.04	0.05
Phosphorylated tau-181	0.11	0.04	0.004^a^	0.04	0.19
Total tau (pg/mL)	0.94	0.15	0.000^a^	0.65	1.23
PTau181/Aβ42	0.02	0.01	0.004^a^	0.01	0.03
Total tau/Aβ42	0.14	0.27	0.000^a^	0.09	0.19
Chitinase (YKL-40)	0.37	0.1	0.001^a^	0.17	0.57
YKL-40/Aβ42	0.001	0.0003	0.01^a^	0.0001	0.001
Neurofilament-light chain (NfL)	0.72	0.32	0.03	0.06	1.37

CSF orexin associations with cognition (control Aβ42)	β coeff	SE	*p* Value	95% CI lower	Upper
ADAS-cog					
Total score base	0.01	0.005	0.04	0.0001	0.019
Total score 12m	0.0135	0.006	0.01^a^	0.003	0.024
Mini-mental state (MMSE)					
Total score base	−0.0.001	0.002	0.58	−0.005	0.003
12m	−0.01	0.002	0.000^a^	−0.011	−0.004
24m	−0.013	0.003	0.011^a^	−0.011	−0.0001
36m	−0.003	0.159	0.98	−0.32	0.031
California verbal learning					
Short-term verbal mem base	−0.0013	0.0004	0.002^a^	−0.002	−0.001
Short-term verbal mem 12m	−0.002	0.0001	0.02^a^	−0.0024	−0.0002
Long-term verbal mem base	−0.0015	0.001	0.08	−0.003	0.0002
Long-term verbal mem 12m	−0.001	0.001	0.2^a^	−0.003	0.001
Rey-Osterrieth					
Long-term visual memory base	−0.0007	0.002	0.76	−0.005	0.004
Long-term visual memory 12m	−0.004	0.0017	0.01^a^	−0.008	−0.0009
Copy-figure test base	0.003	0.003	0.43	−0.004	0.009
Copy-figure test base	−0.002	0.002	0.45	−0.007	0.003
Neuropsychiatric symptom index (NPI)					
base	−0.018	0.008	0.02^a^	−0.034	−0.002
12m	0.03	0.007	0.001^a^	0.011	0.041

Sleep oscillations associations CSF orexin	β coeff	SE	*p* Value	95% CI low	Upper
Sleep spindles (SP)					
Density (/30 s)	−187.37	80.39	0.02^a^	−344.93	−29.80
Duration (s)	−635.14	308.9	0.04	−1,240.76	−29.52
Power (μV^2^)	−0.188	0.08	0.02^a^	−0.04	−0.02
Peak-to-peak amplitude (μV)	−1.35	1.00	0.182	−3.33	0.63
Slow oscillation (SO)					
Density (/30 s)	−20.36	21.92	0.353	−63.33	22.61
Duration (s)	−796.43	215.24	0.000^a^	−1,218.3	−374.56
Power (μV^2^)	0.033	0.019	0.086	−0.01	0.071
Peak-to-peak amplitude (μV)	0.51	0.48	0.293	−0.44	1.46

Abbreviations: AD = Alzheimer disease; ADAS-Cog = Alzheimer's Disease Assessment Scale–Cognitive Subscale; GLM = generalized linear model; SE = sleep efficiency; SO = slow oscillation; SP = sleep spindles.

Units: Aβ42 and pTau181 in picograms per milliliter (pg/mL), SP and SO duration: seconds (s), density: count/30 s, power: microvolts squared (μV^2^), peak-to-peak amplitude: microvolts (μV).

a Statistically significant after controlling for multiple comparisons with Benjamini-Hochberg false discovery rate.

### Orexin and Cognitive or Neuropsychiatric Outcomes

**Figure 2:**
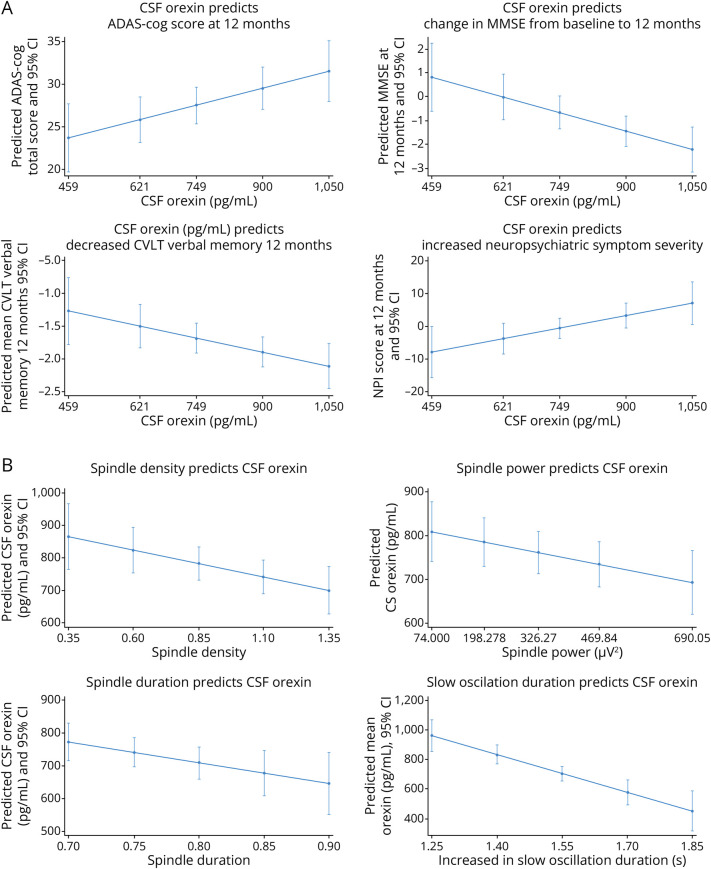
Linear Relationships Between (A) CSF Orexin at Baseline and Cognition at 12 Months and (B) Spindle Density, Power, and Duration, and Slow Oscillation Duration and CSF Orexin ADAS-Cog = Alzheimer's Disease Assessment Scale–Cognitive Subscale; CVLT = California verbal learning test; MMSE = Mini-Mental State Examination; NPI = neuropsychiatric symptoms.

Higher baseline CSF orexin was associated with poorer cognition and greater neuropsychiatric symptom severity over 36 months, whereas stronger SP and SO activity was associated with better performance (Table 2, Figure 2). Higher orexin concentration was associated with higher ADAS-Cog and lower MMSE scores (indicating worse cognitive performance), poorer verbal learning and memory (CVLT), reduced long-term visual memory (ROCF), and worsening NPI scores over time (Table 2, Figure 2). Higher orexin concentrations were also associated with higher concentrations of CSF biomarkers of neurodegeneration and neuroinflammation, including pTau181, total tau, total tau/Aβ42, pTau181/Aβ42, YKL-40, and YKL-40/Aβ42.

### Moderation Analyses

Significant interactions were observed between orexin, NREM sleep oscillatory activity (SP and SO), cognition, and neuropsychiatric symptoms, even after adjusting for Aβ42 and pTau181 (Tables 3 and 4, eTables 2 and 3). Higher orexin concentrations attenuated, but did not abolish, the beneficial effects of SP and SO activity on clinical outcomes (eTable 2). For example, orexin dampened the positive associations of SP density and duration with ADAS-Cog at baseline and 12 months and weakened the relationships between SP power, SP density, SO duration, and SO amplitude and MMSE performance across follow-up. For neuropsychiatric outcomes, higher orexin moderated the protective effects of SP activity on NPI scores, attenuating associations of SP duration and density with reduced symptom severity.

**Table 3:** Statistically Significant Interactions: Sleep Spindles Moderate the Effect of Orexin on Cognition and Neuropsychiatric Symptom Severity (NPI), Controlling for PTau181 and Aβ42

Interactions: spindle activity moderates the effect of orexin on cognition					
Alzheimer's disease assessment scale–cognitive subscale (ADAS-cog): total score	Coeff	SE	*p* Value	95% CI low	Upper
Spindle (SP)					
ADAS-cog base, SP density and Aβ42	0.03	0.013	0.03^a^	0.003	0.05
ADAS-cog base, SP power and Aβ42	0.0003	0.000	0.02^a^	0.00003	0.0005
ADAS-cog base, SP peak-to-peak amplitude and Aβ42	0.001	0.000	0.008^a^	0.0003	0.002
ADAS-cog base, SP density and pTau181	0.25	0.012	0.02^a^	0.003	0.048
ADAS-cog base, SP duration and pTau181	0.11	0.049	0.02^a^	0.01	0.21
ADAS-cog base, SP power and pTau181	0.0003	0.000	0.009^a^	0.0001	0.0004
ADAS-cog base, SP peak-to-peak amplitude and pTau181	0.001	0.000	<0.001^a^	0.0003	0.001
ADAS-cog 12 mo, SP duration and Aβ42	0.09	0.05	0.049^a^	0.0002	0.18
ADAS-cog 12 mo, SP peak-to-peak amplitude, Aβ42	0.001	0.000	0.004^a^	0.0002	0.001
ADAS-cog 12 mo spindle (SP) density and pTau181	0.02	0.01	0.006^a^	0.01	0.04
ADAS-cog 12 mo SP duration and pTau181	0.14	0.05	0.009^a^	0.04	0.24
ADAS-cog 12 mo SP peak-to-peak amplitude, pTau181	0.001	0.000	<0.001^a^	0.0003	0.001
ADAS-cog 12 mo, SO density and Aβ42	−0.01	0.006	0.05	−0.02	0.000
ADAS-cog base 12 mo, SO duration and Aβ42	0.17	0.58	0.005^a^	0.51	0.28
ADAS-cog base 12 mo, SO density and pTau181	−0.22	0.007	0.002^a^	−0.04	−0.009
ADAS-cog base 12 mo, SO duration and pTau181	0.23	0.05	<0.001^a^	0.12	0.35

Mini mental state examination (MMSE)	Coeff	SE	*p* Value	95% CI low	Upper
Spindle (SP)					
MMSE base, SP density and Aβ42	−0.013	0.005	0.016^a^	−0.02	−0.002
MMSE base, SP power and Aβ42	−0.0001	0.000	0.019^a^	−0.0001	−0.0005
MMSE base, SP peak-to-peak amplitude and Aβ42	−0.0002	0.000	0.02^a^	−0.0005	−0.0003
MMSE base SP density and pTau181	−0.02	0.01	0.001^a^	−0.02	−0.01
MMSE 12 mo, SP density and Aβ42	−0.01	0.004	0.007^a^	−0.02	−0.003
MMSE 12 mo, SP duration and Aβ42	0.11	0.05	0.028^a^	0.01	0.21
MMSE 12 mo, SP power and Aβ42	0.0003	0.000	0.04^a^	0.000	0.0001
MMSE 12 mo, SP density and pTau181	−0.013	0.005	0.016^a^	−0.02	−0.002
MMSE 12 mo, SP power and pTau181	−0.0001	0.000	0.019^a^	−0.0001	−0.0005
MMSE 12 mo, SP peak-to-peak amplitude and pTau181	−0.0002	0.000	0.02^a^	−0.0005	−0.0003
MMSE 24 mo, SP density and Aβ42	0.04	0.01	0.001^a^	0.02	0.06
MMSE 24 mo, SP duration and Aβ42	0.19	0.07	0.015^a^	0.37	0.34
MMSE 24 mo, SP power and Aβ42	0.0003	0.000	0.001^a^	0.0001	0.0004
MMSE 24 mo, peak-to-peak amplitude and Aβ42	0.0004	0.000	<0.001^a^	−0.001	−0.0002
MMSE 24 mo, SP density and pTau181	−0.013	0.005	0.016^a^	−0.02	−0.002
MMSE 24 mo, SP power and pTau181	−0.0001	0.000	0.019^a^	−0.0001	−0.0005
MMSE 24 mo, peak-to-peak amplitude, pTau181	−0.0002	0.000	0.02^a^	−0.0005	−0.0003
MMSE 36 mo, SP density and Aβ42	−0.04	0.01	0.010^a^	−0.07	−0.01
MMSE 36 mo, SP duration and Aβ42	−0.51	0.11	<0.001^a^	−0.73	−0.29
MMSE 36 mo, SP power and Aβ42	−0.0004	0.000	<0.001^a^	−0.001	−0.0002
MMSE 36 mo, peak-to-peak amplitude and Aβ42	−0.001	0.000	0.04^a^	−0.001	−0.0003
MMSE 36 mo, SP density and pTau181	−0.02	0.01	0.02^a^	−0.05	−0.003
MMSE 36 mo, SP duration and pTau181	−0.38	0.18	0.031^a^	−0.72	−0.03
MMSE 36 mo, SP power and pTau181	−0.0004	0.000	0.007^a^	−0.001	−0.0001
MMSE 36 mo, peak-to-peak amplitude pTau181	−0.001	0.000	0.04^a^	−0.00	−0.001

Abbreviations: SE = sleep efficiency; SO = slow oscillation.

Units: Aβ42 and pTau181 in picograms per milliliter (pg/mL), SP and SO duration: seconds (s), density: count/30 s, power: microvolts squared (μV2), peak-to-peak amplitude: microvolts (μV).

a Statistically significant interaction (*p* < 0.05).

**Table 4:** Statistically Significant Interactions: Slow Oscillations Moderate the Effect of Orexin on Cognition and Neuropsychiatric Symptom Severity (NPI), Controlling for PTau181 and Aβ42

Interactions: slow oscillation (SO) activity moderates the effect of orexin on cognition					
Alzheimer's disease assessment scale–cognitive subscale (ADAS-cog): total score	Coeff	SE	*p* Value	95% CI low	Upper
ADAS-cog base, SO density and Aβ42					
ADAS-cog base, SO density and pTau181	0.18	0.086	0.036^a^	0.01	0.35
ADAS-cog base, SO duration and pTau181	−0.02	0.004	<0.001^a^	−0.03	−0.01
ADAS-cog base, SO power and pTau181	−0.0001	0.000	<0.001^a^	−0.0001	−0.000
ADAS-cog base, SO peak-to-peak amplitude and pTau181	−0.0002	0.001	0.02^a^	−0.001	−0.000
ADAS-cog 12 mo, SO density and Aβ42	−0.01	0.006	0.05	−0.02	0.000
ADAS-cog base 12 mo, SO duration and Aβ42	0.17	0.58	0.005^a^	0.51	0.28
ADAS-cog base 12 mo, SO density and pTau181	−0.22	0.007	0.002^a^	−0.04	−0.009
ADAS-cog base 12 mo, SO duration and pTau181	0.23	0.05	<0.001^a^	0.12	0.35
Slow oscillation (SO)					
MMSE base, SO duration and Aβ42	0.17	0.58	0.005^a^	0.51	0.28
MMSE base, SO duration and pTau181	0.17	0.58	0.005^a^	0.51	0.28
MMSE 12 mo, SO duration and pTau181	0.17	0.58	0.005^a^	0.51	0.28
MMSE 24, SO power and Aβ42	0.0002	0.000	0.027^a^	0.0003	0.0001
MMSE 24 mo, SO density and pTau181	0.01	0.004	0.03^a^	0.001	0.01
MMSE 24 mo, SO duration and pTau181	−0.09	0.043	0.031^a^	−0.18	−0.01
MMSE 36 mo, SO power and Aβ42	0.0002	0.000	0.013^a^	0.00004	0.0003
MMSE 36 mo, SO peak-to-peak amplitude Aβ42	−0.002	0.000	<0.001^a^	−0.003	−0.001
MMSE 36 mo, SO density and pTau181	0.08	0.013	<0.001^a^	0.059	0.011
MMSE 36 mo, SO power and pTau181	0.0002	0.000	0.007^a^	0.0001	0.0004
MMSE 36 mo, SO peak-to-peak amplitude and pTau181	−0.001	0.0003	0.021^a^	−0.001	−0.0001
Neuropsychiatric symptom index (NPI)					
NPI 12 mo and Aβ42					
NPI 12 mo, SP density and Aβ42	−0.04	0.17	0.01^a^	−0.08	−0.01
NPI 12 mo, SP duration and Aβ42	0.23	0.11	0.03^a^	0.02	0.44
NPI 12 mo, SP density and pTau181	0.07	0.021	0.001^a^	0.03	0.12
NPI 12 mo, SP duration and pTau181	0.28	0.091	0.003^a^	0.10	0.45
NPI 12 mo, SP peak-to-peak amplitude and pTau181	0.001	0.000	0.04^a^	0.000	0.001
Slow oscillation (SO)					
NPI Base, SO duration and Aβ42	−0.240	0.11	0.03^a^	−0.45	−0.26
NPI 12 mo, SO density and pTau181	−0.04	0.012	0.003^a^	−0.05	−0.01
NPI 12 mo, SO duration and pTau181	−0.028	0.12	0.01^a^	−0.51	−0.5
NPI 12 mo, SO power and pTau181	−0.0001	0.000	0.037^a^	−0.001	−0.000

Abbreviation: MMSE = Mini-Mental State Examination; SE = sleep efficiency; SO = slow oscillation.

Units: Aβ42 and pTau181 in picograms per milliliter (pg/mL), SP and SO duration: seconds (s), density: count/30 s, power: microvolts squared (μV^2^), peak-to-peak amplitude: microvolts (μV).

a Statistically significant interaction (*p* < 0.05).

### Reciprocal Buffering Effects of NREM Oscillations

**Figure 3:**
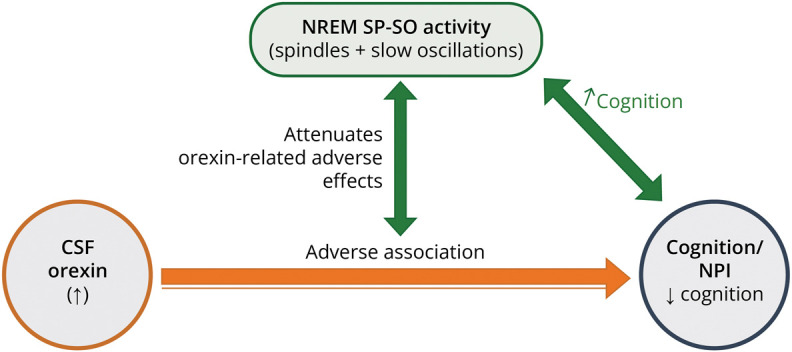
CSF Orexin, NREM SP-SO Activity, and Cognition: Bidirectional Relationships and Moderating Effects NPI = neuropsychiatric symptoms; NREM = nonrapid eye movement; SO = slow oscillation; SP = sleep spindle.

Conversely, multiple SP and SO features mitigated the detrimental effects of higher orexin concentrations on cognition and behavior (Tables 3 and 4, eTable 1). Greater spindle activity attenuated orexin-related decline in global cognition (ADAS-Cog and MMSE), verbal memory (CVLT), visual memory (ROCF), and NPI scores across baseline and longitudinal follow-up. Similarly, longer SO duration and higher SO amplitude buffered against orexin-related worsening of ADAS-Cog and MMSE scores, whereas higher SO density and amplitude reduced orexin-related impairment in visual memory (ROCF) at 12 months. These interactions suggest that NREM oscillations confer resilience to orexin-related decline (Figure 3).

Additional sensitivity analyses adjusting for CSF YKL-40 yielded materially unchanged interaction effects for baseline and 12-month ADAS-Cog and for baseline, 12-, 24-, and 36-month MMSE. YKL-40 was not independently associated with cognitive outcomes in these models. Sensitivity analyses adjusting for OSA severity (severe vs nonsevere; AHI ≥30 events/hour) yielded materially unchanged results, with consistent direction and magnitude of associations between CSF orexin, NREM oscillatory activity, and cognitive and neuropsychiatric outcomes, including moderation effects.

## Discussion

In people with biomarker-confirmed, mild-to-moderate AD, higher CSF orexin concentrations were associated with poorer cognitive performance and greater neuropsychiatric symptom severity over 36 months. Stronger NREM SP and SO activity was associated with better performance and attenuated these adverse associations, even after accounting for Aβ42 and pTau181 (Figure 3). Spindle and SO features were themselves also associated with lower orexin concentrations, and orexin did not relate to global sleep metrics. Together, these findings identify a physiologically and clinically relevant coupling between orexinergic tone and NREM sleep microarchitecture that maps onto longitudinal cognitive and behavioral trajectories in people with AD, through mechanisms largely independent of overall sleep duration. However, in the absence of a cognitively unimpaired comparator group, these findings should be interpreted as reflecting associations within biomarker-confirmed AD, rather than evidence of AD-specific abnormalities relative to normal aging.

Higher CSF orexin concentration was associated with greater decline on global cognition measures (ADAS-Cog, MMSE) and domain-specific memory tests (CVLT, Rey-Osterrieth) and worsening neuropsychiatric symptom severity (NPI). This pattern aligns with orexin's established role in arousal and affective regulation, supporting its contribution to behavioral disturbances in people with AD.^13^ To the best of our knowledge, this is the first longitudinal evidence linking baseline CSF orexin to cognitive and neuropsychiatric symptom severity in biomarker-confirmed AD, extending earlier cross-sectional findings in preclinical and mixed cohorts where AD pathology was absent or uncertain.^18^

Multiple neurobiological pathways could help explain the observed associations between elevated orexin and cognitive and behavioral decline in people with AD. Elevated orexin may contribute to cognitive and behavioral decline through arousal-regulation, sleep stability, and tau-related processes.^6,8,11^ Although experimental studies suggest that sleep disruption may impair glymphatic clearance of amyloid and tau, we did not directly assess glymphatic function in this study. Accordingly, these mechanisms remain speculative and warrant further investigation.

Although orexin is a key regulator of REM sleep, and elevated CSF orexin has been associated with REM sleep disruption in mild cognitive impairment because of AD,^16^ REM sleep metrics were not the primary focus of the present study. Our analyses were designed to specifically examine NREM sleep microarchitecture, given the established roles of sleep spindles and slow oscillations in memory consolidation and neurodegeneration. Nevertheless, REM sleep disruption, including alterations in REM duration and fragmentation, may represent an additional pathway linking orexinergic dysregulation to cognitive and neuropsychiatric outcomes in AD and warrants investigation in future studies.^16^

Orexin was also associated with tau-related and other neurodegeneration biomarkers in our sample. Although evidence linking orexin to AD pathology remains mixed, our results extend prior work by demonstrating these relationships in symptomatic AD with longitudinal clinical follow-up. CSF orexin concentrations in our cohort were higher than values typically reported in cognitively healthy older adults.^41^ Although only a few prior studies or meta-analysis report higher CSF orexin in persons with mild-to-moderate AD^15,18^ compared with healthy controls,^15,42^ converging evidence from animal models and pharmacologic studies implicates orexin in amyloid accumulation.^7,43^ Conversely, other work proposes that increased orexin reflects a compensatory response to amyloid deposition.^44^

In our study, higher CSF orexin was significantly associated with total tau, pTau181, the pTau181/Aβ42 ratio, YKL-40, an established marker of astroglial activation and neuroinflammation, and YKL-40/Aβ42. These findings support the concept that orexinergic changes co-occur with AD-related neurodegenerative and neuroinflammatory markers. Elevated orexin may represent both an upstream driver and a downstream consequence of AD pathology, consistent with experimental data showing that orexin enhances amyloid production and tau phosphorylation while promoting wake-related neuronal activity.^7,43^ Collectively, these findings position orexin as a dynamic biomarker and mechanistic contributor to AD progression.^18,45^ Orexin can be quantified reliably using established CSF assays, suggesting that orexin, alongside Aβ and tau, could enhance clinical phenotyping of sleep-related disease trajectories and identify patients who may benefit from orexin-modulating interventions.

Greater SP activity (density, duration, and power) and SO duration were associated with lower orexin levels, whereas broad sleep continuity measures (TST, sleep efficiency, wake after sleep onset, and sleep-onset latency) showed no associations with orexin. This specificity indicates that orexin's influence in persons with AD relates more directly to NREM sleep microarchitecture than to general sleep quantity or continuity, highlighting SP-SO activity as an active interface through which orexinergic signaling modulates cognition and behavior in people with AD.

The relationships among orexin, SP and SO activity, and neurodegeneration are complex and likely bidirectional.^14^ Slow wave activity has been linked to metabolite clearance in experimental models, and age-related alterations in SP-SO activity have been associated with greater medial prefrontal Aβ burden in cognitively healthy older adults.^46^ Consistent with this broader literature, we found that both SP and SO activity attenuated the adverse associations of higher orexin concentrations with multiple measures of cognitive performance in our sample of people with AD. Notably, the SP/SO moderation effects remained significant after adjustment for CSF YKL-40, suggesting that these associations were not explained by astrocytic activation as indexed by this biomarker. Our previous work has also shown that both SP and SO activity mediate and moderate associations between AD biomarkers and cognition, reinforcing their potential role as physiologic substrates of resilience in people with AD.^20^ However, glymphatic function was not directly assessed in this study, and our findings should not be interpreted as evidence of specific mechanistic pathways.

Intriguingly, orexin levels differed between men and women, despite similar sleep continuity measures. Sex was included as a covariate in all models; however, formal sex-by-orexin interaction analyses were not performed, and the study was not powered to detect effect modification by sex. However, women had higher levels of SP activity than men in our sample. The observed sex difference may reflect hormonal influences and differential vulnerability to tau pathology.

Given the mean age of the cohort, women were likely postmenopausal, and estrogen depletion may contribute to alterations in orexin regulation. Experimental and clinical studies suggest that estrogen modulates orexin neurons and its decline after menopause has been associated with higher orexin levels in women.^47^ Orexin levels can also increase with tau pathology in people with AD.^5,9^ Women in our cohort had higher total-tau and pTau181 levels than men. Although these differences were not statistically significant, they could mechanistically contribute to higher orexin concentrations in women in our sample, despite their higher SP activity. These findings raise the possibility of a sex-specific orexin-tau-sleep interaction that may partly underlie differential AD vulnerability, with potential implications for dose selection, patient stratification, and target engagement assessment in orexin-targeting trials. Although our study was not powered to formally test sex-by-orexin interaction effects, the observed differences underscore the importance of incorporating sex as a biological variable in future mechanistic and interventional studies.

Our moderation analyses indicate that the clinical effect of orexinergic signaling in people with AD depends critically on the integrity of NREM sleep oscillations. Higher CSF orexin was associated with worsening neuropsychiatric symptoms (*β* = 0.040, *p* = 0.005), but this effect was reversed at higher SP density, reflecting a significant interaction (*β* = −0.042, *p* = 0.011). Similar crossover effects were observed for cognition, whereby SP and SO activity mitigated orexin's associations with ADAS-Cog decline and MMSE performance across follow-up. These findings indicate that NREM oscillations not only forecast trajectories of cognitive and neuropsychiatric change but also buffer against the cortical and behavioral consequences of dysregulated orexinergic signaling. NREM oscillations thus represent a potential physiologic marker of resilience and a candidate target for interventions designed to enhance sleep-dependent neural stability in people with AD.

This study has several methodological strengths. Data were prospectively collected using standardized protocols in a sex-balanced cohort with biomarker-confirmed mild-to-moderate AD. Unlike most prior work, which have been cross-sectional, we performed longitudinal cognitive assessments over 3 years. Participants underwent full-night PSG with rigorous exclusion of medications known to affect sleep microarchitecture. CSF orexin-A was collected the morning after PSG, ensuring temporal proximity of key measures. Models were adjusted for major covariates, including age and sleep apnea, and false discovery rate correction reduced the likelihood of type I error. Participants were comprehensively phenotyped, met standard diagnostic criteria, and had cognitive scores and CSF biomarker profiles consistent with mild-to-moderate AD.

Although the sample size was modest, it reflects the rarity of cohorts combining biomarker-confirmed AD, full-night PSG, and CSF sampling within the same week. The consistency of associations across multiple oscillatory metrics supports the robustness of our findings.

Limitations include the observational design, precluding causal inference, and the absence of a cognitively healthy control group. As such, we cannot determine whether observed associations between orexin, NREM microarchitecture, and clinical outcomes are specific to AD or reflect broader aging or neurodegeneration processes. This study was designed to characterize longitudinal clinical trajectories within biomarker-confirmed AD, however. Future studies incorporating age-matched control and other dementia subtypes are needed to establish disease specificity and generalizability.

A high burden of OSA was observed in this cohort, with approximately 70% of participants exhibiting moderate-to-severe disease and 45% meeting criteria for severe OSA (AHI ≥30). This is consistent with prior reports in clinic-based AD populations but has important implications for interpretation. OSA is known to independently alter sleep microarchitecture, including reductions in spindle density, fragmentation of slow-wave activity, and disruption of sleep-dependent physiologic processes implicated in neurodegeneration. Although all analyses adjusted for AHI as a continuous covariate, and sensitivity analyses accounting for OSA severity yielded materially unchanged results, residual confounding related to OSA severity cannot be fully excluded. It is important that orexin was not associated with global sleep continuity measures, and the observed relationships with NREM oscillatory activity persisted after adjustment, suggesting that these findings are not solely driven by sleep-disordered breathing. Nevertheless, future studies incorporating stratified or interventional designs will be important to disentangle the independent and interactive effects of OSA and NREM oscillatory physiology in AD.

PSG was conducted only at baseline and without an adaptation night; therefore, first-night effects cannot be excluded. However, repeated in-lab recordings are challenging in people with AD. The cohort's relatively short TST (mean 260 minutes) reflects sleep fragmentation common in clinic-based AD cohorts and reported previously.^1,5,48^ Although the absence of an adaptation night may also have influenced sleep duration, sensitivity analyses indicated that primary associations between orexin, NREM oscillatory activity, and clinical outcomes were not driven by extremely short sleepers.

Finally, the lack of longitudinal PSG precludes assessment of dynamic changes in sleep physiology relative to biomarker progression. Future studies incorporating longitudinal PSG and CSF orexin measures could clarify the temporal dynamics of orexin-sleep interactions and their utility as therapeutic targets.

Our findings delineate the complex interplay between orexin, sleep microarchitecture, and neurodegeneration in people with AD. Higher orexin concentration was associated with greater cognitive decline, whereas preserved NREM oscillations conferred resilience, attenuating these effects. These results highlight SP, SO, and orexin as complementary physiologic and molecular targets for intervention in people with AD and provide novel physiologic endpoints for ongoing DORA trials in dementia.

Although DORAs improve sleep continuity in insomnia and are being explored in AD-related populations, the directionality of orexin changes in AD remains uncertain.^14^ Orexin deficiency in narcolepsy is associated with sleep-wake instability and fragmented sleep, raising the possibility that excessive orexin suppression could have unintended consequences if higher orexin concentrations in AD reflect, in part, a compensatory response to neurodegeneration or circadian disruption.^6^ Accordingly, future interventional studies should carefully evaluate dose, timing, and patient selection and incorporate physiologic endpoints (e.g., SP/SO metrics) to confirm target engagement and avoid worsening sleep architecture. In parallel, strategies aimed at enhancing NREM oscillatory activity and sleep stability through nonorexin pathways may represent complementary or alternative approaches to support cognitive outcomes in AD.

Identifying such markers is clinically important given the limited availability of disease-modifying therapies. Quantifying NREM oscillatory activity through polysomnography and measuring CSF orexin can provide a feasible framework for integrating sleep physiology into clinical phenotyping and disease monitoring.

An important consideration is whether prognostic validity demonstrated within a single-cohort AD sample, in the absence of a non-AD comparator group, is sufficient to justify the use of orexin and NREM oscillatory metrics as clinical trial endpoints. One perspective is that biomarkers demonstrating longitudinal associations with cognitive and neuropsychiatric trajectories within a cohort with biomarker-confirmed AD may serve as meaningful indicators of disease progression, consistent with biomarker qualification frameworks and precision-medicine approaches in AD research.^49,50^ Alternatively, establishing AD-specific characteristics relative to age-matched controls may be necessary to determine whether these measures reflect disease mechanisms rather than the normal aging processes in line with biological definitions of AD that emphasize disease specificity.^51^ Future longitudinal case-control studies incorporating age- and sex-matched cognitively unimpaired participants will be essential to determine whether orexin and NREM microarchitecture alterations are AD-specific, refine mechanistic models, and inform patient selection and endpoint development in intervention trials.

The EEGs processing, spindles, and slow oscillations detection pipeline is open access and freely available.^30^

## Glossary

AD: Alzheimer disease
ADAS-Cog: Alzheimer’s Disease Assessment Scale–Cognitive Subscale
CVLT: California verbal learning test
DORA: dual orexin receptor antagonist
FDR: false discovery rate
MMSE: Mini-Mental State Examination
NREM: nonrapid eye movement
OSA: obstructive sleep apnea
PSG: polysomnography
pTau181: phosphorylated tau181
RMS: root mean square
SE: sleep efficiency
SO: slow oscillation
TST: total sleep time
VIF: variance inflation factor
